# Filamentous fungi from extreme environments as a promising source of novel bioactive secondary metabolites

**DOI:** 10.3389/fmicb.2015.00903

**Published:** 2015-09-09

**Authors:** Renato Chávez, Francisco Fierro, Ramón O. García-Rico, Inmaculada Vaca

**Affiliations:** ^1^Facultad de Química y Biología, Departamento de Biología, Universidad de Santiago de ChileSantiago, Chile; ^2^División de Ciencias Biológicas y de la Salud, Departamento de Biotecnología, Universidad Autónoma Metropolitana-Unidad IztapalapaMéxico D.F., Mexico; ^3^Grupo GIMBIO, Facultad de Ciencias Básicas, Departamento de Microbiología, Universidad de PamplonaPamplona, Colombia; ^4^Facultad de Ciencias, Departamento de Química, Universidad de ChileSantiago, Chile

**Keywords:** natural products, filamentous fungi, secondary metabolites, extreme environments, genome mining, metagenomics

## Abstract

Natural product search is undergoing resurgence upon the discovery of a huge previously unknown potential for secondary metabolite (SM) production hidden in microbial genomes. This is also the case for filamentous fungi, since their genomes contain a high number of “orphan” SM gene clusters. Recent estimates indicate that only 5% of existing fungal species have been described, thus the potential for the discovery of novel metabolites in fungi is huge. In this context, fungi thriving in harsh environments are of particular interest since they are outstanding producers of unusual chemical structures. At present, there are around 16 genomes from extreme environment-isolated fungi in databases. In a preliminary analysis of three of these genomes we found that several of the predicted SM gene clusters are probably involved in the biosynthesis of compounds not yet described. Genome mining strategies allow the exploitation of the information in genome sequences for the discovery of new natural compounds. The synergy between genome mining strategies and the expected abundance of SMs in fungi from extreme environments is a promising path to discover new natural compounds as a source of medically useful drugs.

## Introduction

The last decades of the 20th century witnessed a decline in the search for natural compounds as pharmaceutically useful drugs. A number of reasons accounted for this trend (Strohl, 2000). However, natural products present key advantages over synthesized chemicals which make them an unsubstitutable source of potential new drugs. They show greater structural diversity as compared to chemical synthetic compounds. This diversity makes them a potentially infinite source of chemical diversity, and as a consequence they present a wide range of biological activities (Demain, 2014). Natural products or compounds derived from them constituted over 35% of approved and pre-new drug application candidates in the period spanning 1981–2010, and 48.6% in the case of anti-cancer drugs since the 1940s (Newman and Cragg, 2012).

Out of about 1 million natural products, ∼25% are biologically active. Of these, about 60% come from plants, and most of the rest from microbes. In the microbial world, fungi stand out as the most prolific producers of bioactive compounds, with ∼42% of the total (Demain, 2014).

Classical methodologies for the discovery of microbial natural products mostly relied in the cultivation of isolates in the laboratory followed by a bioactivity-guided fractionation and identification of the purified compounds. The arrival of the genomic era meant a radical change in the approach to discover new natural products. The analysis of microbial genomes made evident that they contain numerous genes likely to participate in the biosynthesis of structurally complex compounds, but which are not associated with the production of known metabolites. This phenomenon was first recognized in strains from *Streptomyces* (Omura et al., 2001; Bentley et al., 2002) and was soon replicated in filamentous fungi (Keller et al., 2005). Nowadays, it is acknowledged that fungi have a potential of secondary metabolite (SM) production far beyond the number of compounds currently known. Genome mining strategies allow the exploitation of the information provided by genome sequences for the discovery of new natural compounds. This wealth of genomic information is aided by improvements in chemical analytical methodologies, which allow higher separation capacity and sensitivity (Molinski, 2010; Li et al., 2015). Thus a resurgence of natural product discovery is underway, and fungi from extreme environments may make a significant contribution to it.

In this article we will discuss why fungi isolated from extreme environments are an excellent potential source of new natural products with novel and/or unusual chemical structures, and will propose some genome and metagenome mining methodologies which can be successfully applied to them.

## Are Fungi from Extreme Environments Good Potential Producers of Novel Secondary Metabolites?

Each fungal species has its own chemotype, and thus an effective strategy to find new compounds is to study the whole secondary metabolome of new species. The chances of isolating new fungal species are greater if the samples come from non-mesophilic environments, such as those characterized by high salinity, high radiation, limited nutrients, extreme temperatures and pressures and variable acidity. Organisms from these extreme habitats have developed survival strategies for growing and reproducing in such harsh conditions, among them the production of small organic molecules with specific biological activities, for example, cryoprotectant molecules such as sugars and polyols to stabilize membranes and maintain turgor pressure (Timling and Taylor, 2012), osmotically active compounds such as polyols in xerotolerant fungi (Gunde-Cimerman and Zalar, 2014), and fungal melanins for protection against freezing and UV radiation (Nosanchuk and Casadevall, 2003). Since fungi from extreme habitats are subjected to environmental conditions not met by mesophilic fungi, it is expected that many of the compounds they synthesize are specific to them.

Fungi from extreme environments seem therefore good potential candidates for the isolation of new bioactive compounds, and this interest has been reflected in the gradual increase of published articles reporting new compounds from these fungi. Supplementary Table S1 summarizes the compounds isolated from fungi growing in extreme environments reported since 2004. Although the number of studies is still low, a remarkable percentage of them (around 40%) report the isolation of new natural products with unusual structural features, for instance the spiromastixones A–O, a group of depsidone-based derivatives from *Spiromastix* sp. (isolated from deep sea sediments of the South Atlantic Ocean; Niu et al., 2014) which contain *n*-propyl substituents in their structure, a feature rarely found in natural products, and the four asterric acid derivatives bearing the unusual nitro group found in the Antarctic fungus *Pseudogymnoascus* sp. (Figueroa et al., 2015). This percentage compares favorably with what is usually found in mesophilic fungi; in a search we performed on studies published since 2004 describing new compounds from a typical mesophilic SM producer group, *Aspergillus* section Nigri, we found that only in 13% of the studies compounds showing unusual, previously undescribed, structures were reported. Therefore, there seems to be sufficient evidence that fungi isolated from extreme environments have an excellent potential as producers of unusual compounds with unique structures.

## How to Maximize Natural Products Discovery from Extreme Environment Fungi

When considering strategies for the isolation of compounds from fungi growing in extreme environments, several considerations must be taken into account. Firstly, isolates from extreme environments may have individual specific requirements to express their SM pathways which are not easy to find/set in the laboratory; likewise other fungi, many pathways are expected to remain silent under laboratory conditions (see below). Secondly, how many of the total number of fungi living in such environments are we able to cultivate in the laboratory?, and thus how many are unculturable and evade our screenings? Magnuson and Lasure (2002) reported that 70–90% of fungi in different soil environments were unculturable. Genome mining offers an array of methods to face the first problem, whereas metagenomics can be applicable to widen the scope of our search for bioactive fungal compounds to those produced by unculturable fungi. Both approaches will be discussed in the next sections.

Traditionally, isolation of natural compounds produced by fungi was based on culturing them under standard laboratory conditions. Variations in the conditions and the media have great impact on the profile of SMs produced (Larsen et al., 2005), and thus different OSMAC (one strain-many compounds, Bode et al., 2002) strategies are developed by research groups to induce the synthesis of compounds (e.g., VanderMolen et al., 2013; Hewage et al., 2014). In the particular case of fungi from extreme environments, culture conditions different from the standard ones have been sometimes used in an attempt to recreate environmental characteristics of their habitats. Most of fungi isolated from cold environments are cultured at 15–20°C, and fungi isolated from saline environments are grown in media with NaCl concentrations of 10% or above. However, a significant number of the reports in Supplementary Table S1 used standard culture conditions, this is especially noticeable for fungi isolated from deep-sea samples, mostly grown at 28°C and atmospheric pressure. The use of conditions very different from those present in the native environments hinders the prospects of finding new compounds from extreme environment fungi.

The analysis of fungal genomes has revealed that the potential number of SMs fungi can produce is much higher than what can be isolated and identified using classical approaches. The main reason that explains why many SMs “encoded” in the genome are not detected seems to be that SM clusters are often silent when fungi are cultured in the laboratory (Brakhage et al., 2008; Hertweck, 2009), a phenomenon that might be still more extensive in fungi from extreme environments. One of the main challenges of genome mining is how to activate these silent clusters so that the “cryptic” compounds they produce can be isolated. Epigenetic regulation has been pointed as the main mechanism causing SM gene cluster silencing (Reyes-Domínguez et al., 2010; Strauss and Reyes-Domínguez, 2011). A general strategy for activation of silent clusters is the use of heterochromatin remodeling compounds, such as 5-azacytidine (DNA methyltransferase inhibitor), trichostatine A or suberoylanilide hydroxamic acid (histone deacetylase inhibitors). Several reports describe that addition of these inhibitors to culture media causes changes in the expression profile of SM gene clusters (Lin et al., 2013), the profile of metabolites detected in the broths (Williams et al., 2008; Chung et al., 2013), and has allowed the isolation of previously unknown compounds, for example nygerone A from *Aspergillus niger* (Henrikson et al., 2009) and new sesquiterpenoids from *Aspergillus sydowii* (Chung et al., 2013). Since this strategy does not require sequencing of the genome, it may be suitable for initial metabolite screenings of extreme environment fungi, in combination with OSMAC strategies.

## Genome Mining and Its Application to the Discovery of New Metabolites from Extreme Environment-Isolated Fungi

Genome mining requires an extensive amount of genome sequence information in order to explore in-depth the SM biosynthetic potential of a microorganism. Many of the SM gene clusters are expected to remain silent when extreme environment fungi are cultured in the laboratory, and therefore identifying these clusters is a key step to exploit this potential. For new isolates, top–down approaches (OSMAC-based strategies in combination with the use of epigenetic modifiers) can be useful to select the best candidates for more in-depth analysis, and may even result in the production and identification of some interesting compounds (Bode et al., 2002; Wang et al., 2014). Isolates showing the best potential capacity for production of new compounds can then be selected for genome analysis. The increasing number of facilities for whole genome sequencing will allow that more specimens can be analyzed in this manner in a near future.

At present, there are some 515 completed or in progress fungal genomes in public databases, of which around 16 belong to fungi isolated from extreme environments. As a preliminary assessment to test if fungi from extreme environments can be a valuable source of novel SMs we scanned the genomes of three fungi, the cold-loving *Pseudogymnoascus pannorum* and *P. destructans* and the thermophilic *Thermomyces lanuginosus*. We used antiSMASH, a software designed to systematically predict clustered SM genes based on their genomic context and domain content (Blin et al., 2013; Weber et al., 2015). The results of this analysis are summarized in **Table 1**. When compared to a model fungus, *Aspergillus nidulans*, the three analyzed fungi show an interesting potential as producers of novel SMs. They have a similar number of SM clusters (14–20) and, interestingly, no more than three of them (depending on the species) are homologous to previously described clusters with known function, that is, the compound they putatively synthesize has been identified in other organisms. Also remarkable is the fact that although several clusters in the extreme environment fungi present partial synteny with clusters from other genomes, they possess 1–3 clusters that seem to be unique, since they have no significant homology to any other genomic sequence in databases. In comparison, all *A. nidulans* clusters have homologs in other organisms. These unique clusters are likely to be involved in the synthesis of novel compounds, possibly with novel/unusual chemical structures.

**Table 1:** Summary of SM clusters found by antiSMASH in some fungal genomes.

Organism	GenBank accession number of the genome	Environmental characteristic	Genome size (Mbp)	Number of clusters found by antiSmash	Number of clusters homologous to clusters with known function (%)	Number of clusters without homologs in other genomes (%)
*Aspergillus nidulans*	BN001301; BN001302; BN001303; BN001304; BN001305; BN001306; BN001307; BN001308	Mesophilic (model) fungus	31	58	12 (20.7)	0 (0)
*Pseudogymnoascus destructans*	AEFC00000000	Cold-loving fungus	30.5	14	2 (14.3)	1 (7.1)
*Pseudogymnoascus pannorum*	AYKR00000000	Cold-loving fungus	29.5	20	3 (15.0)	2 (10.0)
*Thermomyces lanuginosus*	ANHP00000000	Thermophilic fungus	23.3	17	2 (11.8)	3 (17.6)

There is an important difference in the number of SM clusters found with antiSMASH between the extreme environment fungi and *A. nidulans*. Taking into account that their genome sizes are similar (although that of *T. lanuginosus* is 23% smaller than the *A. nidulans* one), this difference is somewhat striking. Biological differences might account for this discordance, they are all Ascomycota, but from different lineages. *Pseudogymnoascus* spp. belongs to the class Leotiomycetes, whereas *A. nidulans* and *T. lanuginosus* are Eurotiales. Another plausible explanation is that results are biased by the way of working of antiSMASH. This software compares the predicted proteins in a DNA sequence against a curated collection of profile hidden Markov models (pHMM) that describe sequences of key biosynthetic enzymes of known classes of SMs. Since pHMM are probabilistic models built from an aligned set of representative sequences, these models can be biased by the sequences used in their construction. Genomes from mesophilic fungus such *A. nidulans* are the most abundant in databases, and therefore, they were probably intensively used in the generation of these pHMM. Therefore, analyses performed with these pHMM will easily find clusters showing similarity to those from mesophilic organisms, but may be less proficient to find novel enzymes of novel SM pathways from extreme environment fungi. Other bioinformatics tools for SM gene search are also available (Khaldi et al., 2010; Fedorova et al., 2012; Boddy, 2014), and the combined capacities of these tools may yield improved results.

Once a new SM cluster has been identified, the next step is isolating and identifying the metabolite/s it synthesizes, this is one of the bottlenecks of genome mining. The structure of the cluster, the filiation of the genes and the domains present in the encoded enzymes may offer clues about the chemical features of the corresponding metabolite, and thus directed separation and analytical strategies can be applied for its identification, an approach that has yielded successful results for the isolation of aspoquinolones A–D and PKS-NRPS hybrid compounds from *A. nidulans* (Scherlach and Hertweck, 2006; Bergmann et al., 2007). A refinement of this approach is the so-called genomisotopic approach, which makes use of isotope-labeled molecules predicted by bioinformatics to guide the purification process (Gross et al., 2007).

**FIGURE 1:**
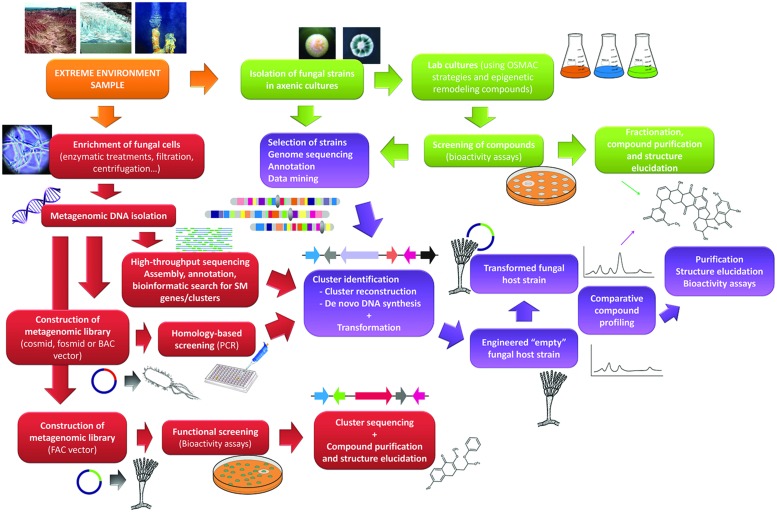
**Flowchart describing the main steps in different approaches to search for new bioactive compounds from extreme environment fungi.** Classical methodologies (green panels) are shown along with genome mining (violet color panels) and metagenomic (garnet color panels) approaches.

A second major bottleneck is the fact that many SM clusters remain silent even when submitted to different culture conditions. Several reviews on genome mining have been published dealing with the issue of activation of silent clusters and identification of their metabolites (Scherlach and Hertweck, 2009; Brakhage and Schroeckh, 2011; Chiang et al., 2014; Luo et al., 2014; Wiemann and Keller, 2014). Basically, the methodologies described can be divided in two groups, (1) those aiming to activate clusters in the host strain, for instance by using genes involved in epigenetic regulation (Strauss and Reyes-Domínguez, 2011) or by overexpressing a specific cluster transcriptional regulator (Bromann et al., 2012), and (2) those making use of heterologous expression. In the case of extreme environment fungi, molecular tools are not readily available to apply most of the strategies lying in the first group, except for the utilization of chromatin remodeling compounds mentioned above. Thus the strategy of choice will be the heterologous expression of the cluster in an appropriate host (**Figure 1**). We enter here in the field of synthetic biology. Different strategies have been developed to reconstruct a whole SM cluster for transferring into a recipient host (reviewed in Lazarus et al., 2014; Anyaogu and Mortensen, 2015). Yeast recombinational cloning has been utilized to clone entire gene clusters and transform *A. nidulans* with them (Yin et al., 2013). Another possibility is the use of fusion PCR to reconstruct a cluster and then transfer it to the host strain (Chiang et al., 2013). This latter work used an engineered *A. nidulans* strain deficient in its own SM clusters, which facilitates the structure elucidation of the heterologous compound. Other modified recipient strains for heterologous expression of SM clusters have recently been developed (Richter et al., 2014; Kakule et al., 2015). The improvement and increasing affordability of techniques for synthesis of long stretches of DNA (Bang and Church, 2008; Carlson, 2009) will facilitate synthesis of entire clusters, including designed clusters with host promoters and regulatory elements for better gene expression.

## Metagenomics for Extreme Environment Fungi in the Search for New Bioactive Compounds

A comprehensive study on the potential of fungi from extreme environments to produce new bioactive compounds would be incomplete if we did not take into account the unculturable fungi. Currently we have no reliable information regarding the percentage of unculturable fungi present in extreme environments, as much as we do not have information about the capacity of these fungi to produce SMs in comparison to the culturable ones. However, the presumed abundance of unculturable fungi in different environments (Magnuson and Lasure, 2002), and the success of some published studies in finding bacterial SM clusters from complex DNA samples and isolating specific metabolites after their heterologous expression (Nikolouli and Mossialos, 2011 and references therein; Owen et al., 2015) point to a hidden wealth of fungal metabolites waiting to be found, and the feasibility to retrieve them.

The use of metagenomes for the discovery of new natural products is receiving considerable attention (Nováková and Farkašovskỳ, 2013; Wilson and Piel, 2013; Charlop-Powers et al., 2014). Metagenomes usually comprise DNA from prokaryotic and eukaryotic organisms, but some strategies can be devised to enrich some particular type. Jiao et al. (2006) used an enzymatic treatment with cellulases to enrich bacterial DNA from plant samples. Enrichment strategies for extreme environment fungi may be developed too. Eukaryotic DNA could be enriched using lysozime and murein hydrolases followed by differential centrifugation, and combined with cellulase treatment fungal DNA may result prevalent. From here the usual approaches to study metagenomes can be followed (**Figure 1**). First the choice of using high-throughput sequencing or constructing metagenomic libraries. In the first case an arduous work of assembly, annotation and bioinformatic analyses follows, but which can produce extensive sequence information to retrieve entire SM cluster sequences. In the second case vectors allowing large DNA inserts (e.g., BACs) would be preferable due to the often big size of SM clusters. Libraries can then be submitted to sequence-based screenings in search for SM clusters. Identified clusters can then be transferred to heterologous hosts as described in the previous section. The recent development of FACs (fungal artificial chromosomes) successfully tested to clone and express entire fungal SM clusters in *A. nidulans* (Bok et al., 2015) is a powerful tool which will allow functional screening of fungal DNA-containing metagenomes.

An interesting approach to estimate the SM biosynthetic potential of different environments was described by Woodhouse et al. (2013), who used TEFAP (Tag-encoded FLX Amplicon Pyrosequencing) targeting NRPS and PKS genes to determine the identity and diversity of SM biosynthetic genes in a complex sample, finding that Australian marine sponges are a good niche to explore for new natural products.

## Conclusion

Fungi gowing in extreme environments are a potential huge reservoir of novel bioactive natural products. Preliminary search of three genomes from extreme environment fungi revealed the presence of several SM clusters with no significant homology to any other genomic sequence in databases or containing only some genes showing identity to others in databases (**Table 1**). These clusters are likely to form novel compounds with unusual structures, a feature previously reported in other extreme environment fungi (Supplementary Table S1). Genome and metagenome mining technologies will make it possible to exploit this valuable resource.

## Conflict of Interest Statement

The authors declare that the research was conducted in the absence of any commercial or financial relationships that could be construed as a potential conflict of interest.

## References

[B1] Anyaogu D. C. Mortensen U. H. (2015). Heterologous production of fungal secondary metabolites in *Aspergilli*. *Front. Microbiol.* 6:77 10.3389/fmicb.2015.00077 PMC4322707 25713568

[B2] Bang D. Church G. M. (2008). Gene synthesis by circular assembly amplification. *Nat. Methods* 5 37–39. 10.1038/nmeth1136 18037891

[B3] Bentley S. D. Chater K. F. Cerdeno-Tarraga A.-M. Challis G. L. Thomson N. R. James K. D. (2002). Complete genome sequence of the model actinomycete *Streptomyces coelicolor* A3(2). *Nature* 417 141–147. 10.1038/417141a 12000953

[B4] Bergmann S. Schuemann J. Scherlach K. Lange C. Brakhage A. A. Hertweck C. (2007). Genomics-driven discovery of PKS-NRPS hybrid metabolites from *Aspergillus nidulans*. *Nat. Chem. Biol.* 3 213–217. 10.1038/nchembio869 17369821

[B5] Blin K. Medema M. H. Kazempour D. Fischbach M. A. Breitling R. Takano E. (2013). antiSMASH 2.0–a versatile platform for genome mining of secondary metabolite producers. *Nucleic Acids Res.* 41 W204–W212. 10.1093/nar/gkt449 23737449 PMC3692088

[B6] Boddy C. N. (2014). Bioinformatics tools for genome mining of polyketide and non-ribosomal peptides. *J. Ind. Microbiol. Biotechnol.* 41 443–450. 10.1007/s10295-013-1368-1 24174214

[B7] Bode H. B. Bethe B. Höfs R. Zeeck A. (2002). Big effects from small changes: possible ways to explore nature’s chemical diversity. *Chembiochem* 3 619–627. 10.1002/1439-7633(20020703)3:7<619::AID-CBIC619>3.0.CO;2-9 12324995

[B8] Bok J. W. Ye R. Clevenger K. D. Mead D. Wagner M. Krerowicz A. (2015). Fungal artificial chromosomes for mining of the fungal secondary metabolome. *BMC Genomics* 16:343 10.1186/s12864-015-1561-x PMC4413528 25925221

[B9] Brakhage A. A. Schroeckh V. (2011). Fungal secondary metabolites - strategies to activate silent gene clusters. *Fungal Genet. Biol.* 48 15–22. 10.1016/j.fgb.2010.04.004 20433937

[B10] Brakhage A. A. Schuemann J. Bergmann S. Scherlach K. Schroeckh V. Hertweck C. (2008). Activation of fungal silent gene clusters: a new avenue to drug discovery. *Prog. Drug Res.* 66 3–12. 10.1007/978-3-7643-8595-8_1 18416304

[B11] Bromann K. Toivari M. Viljanen K. Vuoristo A. Ruohonen L. Nakari-Setala T. (2012). Identification and characterization of a novel diterpene gene cluster in *Aspergillus nidulans*. *PLoS ONE* 7:e35450 10.1371/journal.pone.0035450 PMC3323652 22506079

[B12] Carlson R. (2009). The changing economics of DNA synthesis. *Nat. Biotechnol.* 27 1091–1094. 10.1038/nbt1209-1091 20010582

[B13] Charlop-Powers Z. Milshteyn A. Brady S. F. (2014). Metagenomic small molecule discovery methods. *Curr. Opin. Microbiol.* 19 70–75. 10.1016/j.mib.2014.05.021 25000402 PMC4135586

[B14] Chiang Y. M. Chang S. L. Oakley B. R. Wang C. C. (2014). Recent advances in awakening silent biosynthetic gene clusters and linking orphan clusters to natural products in microorganisms. *Curr. Opin. Chem. Biol.* 15 137–143. 10.1016/j.cbpa.2010.10.011 21111669 PMC3117463

[B15] Chiang Y. M. Oakley C. E. Ahuja M. Entwistle R. Schultz A. Chang S. L. (2013). An efficient system for heterologous expression of secondary metabolite genes in *Aspergillus nidulans*. *J. Am. Chem. Soc.* 135 7720–7731. 10.1021/ja401945a 23621425 PMC3697937

[B16] Chung Y. M. Wei C. K. Chuang D. W. El-Shazly M. Hsieh C. T. Asai T. (2013). An epigenetic modifier enhances the production of anti-diabetic and anti-inflammatory sesquiterpenoids from *Aspergillus sydowii*. *Bioorg. Med. Chem.* 21 3866–3872. 10.1016/j.bmc.2013.04.004 23647825

[B17] Demain A. L. (2014). Importance of microbial natural products and the need to revitalize their discovery. *J. Ind. Microbiol. Biotechnol.* 41 185–201. 10.1007/s10295-013-1325-z 23990168

[B18] Fedorova N. D. Moktali V. Medema M. H. (2012). Bioinformatics approaches and software for detection of secondary metabolic gene clusters. *Methods Mol. Biol.* 944 23–45. 10.1007/978-1-62703-122-6_2 23065606

[B19] Figueroa L. Jiménez C. Rodríguez J. Areche C. Chávez R. Henríquez M. (2015). 3-Nitroasterric acid derivatives from an Antarctic sponge-derived *Pseudogymnoascus* sp. *fungus. J. Nat. Prod.* 78 919–923. 10.1021/np500906k 25732560

[B20] Gross H. Stockwell V. O. Henkels M. D. Nowak-Thompson B. Loper J. E. Gerwick W. H. (2007). The genomisotopic approach: a systematic method to isolate products of orphan biosynthetic gene clusters. *Chem. Biol.* 14 53–63. 10.1016/j.chembiol.2006.11.007 17254952

[B21] Gunde-Cimerman N. Zalar P. (2014). Extremely halotolerant and halophilic fungi inhabit brine in solar salterns around the globe. *Food Technol. Biotechnol.* 52 170–179.

[B22] Henrikson J. C. Hoover A. R. Joyner P. M. Cichewicz R. H. (2009). A chemical epigenetics approach for engineering the in situ biosynthesis of a cryptic natural product from *Aspergillus niger*. *Org. Biomol. Chem.* 7 435–438. 10.1039/B819208A 19156306

[B23] Hertweck C. (2009). Hidden biosynthetic treasures brought to light. *Nat. Chem. Biol.* 5 450–452. 10.1038/nchembio0709-450 19536102

[B24] Hewage R. T. Aree T. Mahidol C. Ruchirawat S. Kittakoop P. (2014). One strain-many compounds (OSMAC) method for production of polyketides, azaphilones, and an isochromanone using the endophytic fungus *Dothideomycete* sp. *Phytochemistry* 108 87–94. 10.1016/j.phytochem.2014.09.013 25310919

[B25] Jiao J. Y. Wang H. X. Zeng Y. Shen Y. M. (2006). Enrichment for microbes living in association with plant tissues. *J. Appl. Microbiol.* 100 830–837. 10.1111/j.1365-2672.2006.02830.x 16553739

[B26] Kakule T. B. Jadulco R. C. Koch M. Janso J. E. Barrows L. R. Schmidt E. W. (2015). Native promoter strategy for high-yielding synthesis and engineering of fungal secondary metabolites. *ACS Synth. Biol.* 4 625–633. 10.1021/sb500296p 25226362 PMC4487227

[B27] Keller N. P. Turner G. Bennett J. W. (2005). Fungal secondary metabolism-from biochemistry to genomics. *Nat. Rev. Microbiol.* 3 937–947. 10.1038/nrmicro1286 16322742

[B28] Khaldi N. Seifuddin F. T. Turner G. Haft D. Nierman W. C. Wolfe K. H. (2010). SMURF: genomic mapping of fungal secondary metabolite clusters. *Fungal Genet. Biol.* 47 736–741. 10.1016/j.fgb.2010.06.003 20554054 PMC2916752

[B29] Larsen T. O. Smedsgaard J. Nielsen K. F. Hansen M. E. Frisvad J. C. (2005). Phenotypic taxonomy and metabolite profiling in microbial drug discovery. *Nat. Prod. Rep.* 22 672–695. 10.1039/b404943h 16311630

[B30] Lazarus C. M. Williams K. Bailey A. M. (2014). Reconstructing fungal natural product biosynthetic pathways. *Nat. Prod. Rep.* 31 1339–1347. 10.1039/C4NP00084F 25140842

[B31] Li K. Chung-Davidson Y.-W. Bussy U. Li W. (2015). Recent advances and applications of experimental technologies in marine natural product research. *Mar. Drugs* 13 2694–2713. 10.3390/md13052694 25939037 PMC4446601

[B32] Lin J. Q. Zhao X. X. Zhi Q. Q. Zhao M. He Z. M. (2013). Transcriptomic profiling of *Aspergillus flavus* in response to 5-azacytidine. *Fungal Genet. Biol.* 56 78–86. 10.1016/j.fgb.2013.04.007 23644151

[B33] Luo Y. Cobb R. E. Zhao H. (2014). Recent advances in natural product discovery. *Curr. Opin. Biotechnol.* 30 230–237. 10.1016/j.copbio.2014.09.002 25260043 PMC4253731

[B34] Magnuson J. K. Lasure L. L. (2002). *Fungal Diversity in Soils as Assessed by Direct Culture and Molecular Techniques.* (Salt Lake: Abstracts from the 102nd General Meeting of the American Society for Microbiology), 19–23. Available at: http://www.pnnl.gov/biobased/docs/fungal_diversity.pdf

[B35] Molinski T. F. (2010). Microscale methodology for structure elucidation of natural products. *Curr. Opin. Biotechnol.* 21 819–826. 10.1016/j.copbio.2010.09.003 20880694 PMC2982861

[B36] Newman D. J. Cragg G. M. (2012). Natural products as sources of new drugs over the 30 years from 1981 to 2010. *J. Nat. Prod.* 75 311–335. 10.1021/np200906s 22316239 PMC3721181

[B37] Nikolouli K. Mossialos D. (2011). Bioactive compounds synthesized by non-ribosomal peptide synthetases and type-I polyketide synthases discovered through genome-mining and metagenomics. *Biotechnol. Lett.* 34 1393–1403. 10.1007/s10529-012-0919-2 22481301

[B38] Niu S. Liu D. Hu X. Proksch P. Shao Z. Lin W. (2014). Spiromastixones A-O, antibacterial chlorodepsidones from a deep-sea-derived *Spiromastix* sp. fungus. *J. Nat. Prod.* 77 1021–1030. 10.1021/np5000457 24571273

[B39] Nosanchuk J. D. Casadevall A. (2003). The contribution of melanin to microbial pathogenesis. *Cell. Microbiol.* 5 203–223. 10.1046/j.1462-5814.2003.00268.x 12675679

[B40] Nováková J. Farkašovskỳ M. (2013). Bioprospecting microbial metagenome for natural products. *Biologia* 68 1079–1086. 10.2478/s11756-013-0246-7

[B41] Omura S. Ikeda H. Ishikawa J. Hanamoto A. Takahashi C. Shinose M.et al. (2001). Genome sequence of an industrial microorganism *Streptomyces avermitilis*: deducing the ability of producing secondary metabolites. *Proc. Natl. Acad. Sci. U.S.A.* 98 12215–12220. 10.1073/pnas.211433198 11572948 PMC59794

[B42] Owen J. G. Charlop-Powers Z. Smith A. G. Ternei M. A. Calle P. Y. Reddy B. V. (2015). Multiplexed metagenome mining using short DNA sequence tags facilitates targeted discovery of epoxyketone proteasome inhibitors. *Proc. Natl. Acad. Sci. U.S.A.* 112 4221–4226. 10.1073/pnas.1501124112 25831524 PMC4394318

[B43] Reyes-Domínguez Y. Bok J. W. Berger H. Shwab E. K. Basheer A. Gallmetzer A. (2010). Heterochromatic marks are associated with the repression of secondary metabolism clusters in *Aspergillus nidulans*. *Mol. Microbiol.* 76 1376–1386. 10.1111/j.1365-2958.2010.07051.x 20132440 PMC2904488

[B44] Richter L. Wanka F. Boecker S. Storm D. Kurt T. Vural Ö. (2014). Engineering of *Aspergillus niger* for the production of secondary metabolites. *Fungal Biol. Biotechnol.* 1 4 10.1186/s40694-014-0004-9 PMC5598268 28955446

[B45] Scherlach K. Hertweck C. (2006). Discovery of aspoquinolones A-D, prenylated quinoline-2-one alkaloids from *Aspergillus nidulans*, motivated by genome mining. *Org. Biomol. Chem.* 4 3517–3520. 10.1039/b607011f 17036148

[B46] Scherlach K. Hertweck C. (2009). Triggering cryptic natural product biosynthesis in microorganisms. *Org. Biomol. Chem.* 7 1753–1760. 10.1039/b821578b 19590766

[B47] Strauss J. Reyes-Domínguez Y. (2011). Regulation of secondary metabolism by chromatin structure and epigenetic codes. *Fungal Genet. Biol.* 48 62–69. 10.1016/j.fgb.2010.07.009 20659575 PMC3935439

[B48] Strohl W. R. (2000). The role of natural products in a modern drug discovery program. *Drug Discov. Today* 5 39–41. 10.1016/S1359-6446(99)01443-9 10652450

[B49] Timling I. Taylor D. L. (2012). Peeking through a frosty window: molecular insights into the ecology of *Arctic soil* fungi. *Fungal Ecol.* 5 419–429. 10.1016/j.funeco.2012.01.009

[B50] VanderMolen K. M. Raja H. A. El-Elimat T. Oberlies N. H. (2013). Evaluation of culture media for the production of secondary metabolites in a natural products screening program. *AMB Express* 3 71 10.1186/2191-0855-3-71 PMC3917616 24342059

[B51] Wang W.-J. Li D.-Y. Li Y.-C. Hua H.-M. Ma E.-L. Li Z.-L. (2014). Caryophyllene sesquiterpenes from the marine-derived fungus *Ascotricha* sp. *ZJ-M*-5 by the one strain–many compounds strategy. *J. Nat. Prod.* 77 1367–1371. 10.1021/np500110z 24878335

[B52] Weber T. Blin K. Duddela S. Krug D. Kim H. U. Bruccoleri R. (2015). antiSMASH 3.0–a comprehensive resource for the genome mining of biosynthetic gene clusters. *Nucleic Acids Res.* 43 W237–W243. 10.1093/nar/gkv437 25948579 PMC4489286

[B53] Wiemann P. Keller N. P. (2014). Strategies for mining fungal natural products. *J. Ind. Microbiol. Biotechnol.* 41 301–313. 10.1007/s10295-013-1366-3 24146366

[B54] Williams R. B. Henrikson J. C. Hoover A. R. Lee A. E. Cichewicz R. H. (2008). Epigenetic remodeling of the fungal secondary metabolome. *Org. Biomol. Chem.* 6 1895–1897. 10.1039/b804701d 18480899

[B55] Wilson M. C. Piel J. (2013). Metagenomic approaches for exploiting uncultivated bacteria as a resource for novel biosynthetic enzymology. *Chem. Biol.* 20 636–647. 10.1016/j.chembiol.2013.04.011 23706630

[B56] Woodhouse J. N. Fan L. Brown M. V. Thomas T. Neilan B. A. (2013). Deep sequencing of non-ribosomal peptide synthetases and polyketide synthases from the microbiomes of Australian marine sponges. *ISME J.* 7 1842–1851. 10.1038/ismej.2013.65 23598791 PMC3749504

[B57] Yin W. B. Chooi Y. H. Smith A. R. Cacho R. A. Hu Y. White T. C. (2013). Discovery of cryptic polyketide metabolites from dermatophytes using heterologous expression in *Aspergillus nidulans*. *ACS Synth. Biol.* 2 629–634. 10.1021/sb400048b 23758576 PMC3795930

