# Supplementary material for: Filamentous fungi from extreme environments as a promising source of novel bioactive secondary metabolites
Source: Front Microbiol. 2015 Sep 9;6:903. doi: 10.3389/fmicb.2015.00903 (PMC4563253; doi:10.3389/fmicb.2015.00903)
Supplement: Supplementary file 1 [file Table_1.DOC]

**
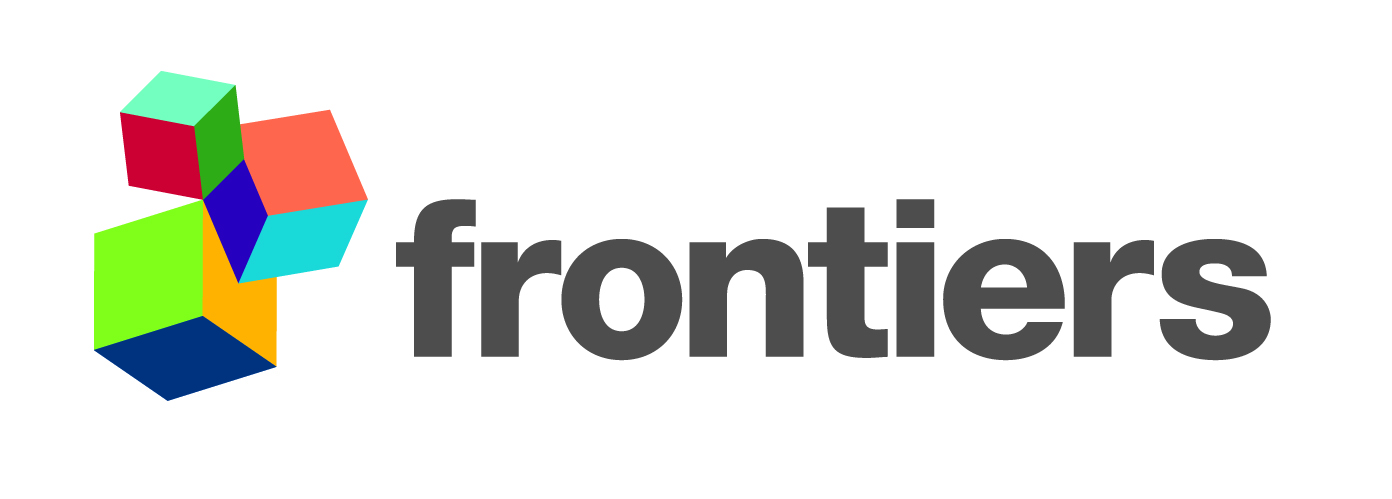
**

***Supplementary Material***

**Filamentous fungi from extreme environments as a promising source of novel bioactive secondary metabolites**

**Renato Chávez^1^, Francisco Fierro^2^*, Ramón O. García-Rico^3^, Inmaculada Vaca^4^***

***Correspondence:** Francisco Fierro, División de Ciencias Biológicas y de la Salud, Departamento de Biotecnología, Universidad Autónoma Metropolitana-Unidad Iztapalapa, Avda. San Rafael Atlixco 186, Colonia Vicentina, México D.F., 09340, México. fierrof@xanum.uam.mx

***Correspondence:** Inmaculada Vaca, Facultad de Ciencias, Departamento de Química, Universidad de Chile, Las Palmeras 3425, Ñuñoa, Santiago, 7800024, Chile. inmavaca@uchile.cl

**Supplementary Table 1**. Chemical compounds isolated from extreme environment fungi

| **Isolated from:** | **Fungal isolate** | **New compounds** | **generic chemical structure** | **reference** |
| --- | --- | --- | --- | --- |
| Sonoran Desert, Arizona, United States | *Aspergillus terreus* | terrequinone A | asterriquinone | He et al., 2004 |
|  |  | terrefuranone | dihydrofuran-4-one |  |
|  | *A. cervinus* | 4R*,5S*-dihydroxy-3-methoxy-5-methylcyclohex-2-enone (7); 6-methoxy-5(6)-dihydropenicillic acid | cyclic-oxygenated-enone structure |  |
|  | *Chaetomium globosum* | globosumones A-C | esters of orsellinic acid | Bashyal et al., 2005 |
| Sihchong River Hot Springs ,Taiwan | *Malbranchea sulfurea* | malbranpyrroles A–F | polyketides | Yang et al., 2009 |
| Tengchong hot springs, Republic of China | *Talaromyces thermophilus* | talathermophilins A-D | prenylated indole  alkaloids | Chu et al., 2010 ; Guo et al., 2011; Guo et al., 2012 |
|  |  | thermolides A−F | macrolactones |  |
| Greenland | *Penicillium rivulum* | psychrophilin B and C | cyclic nitropeptides | Dalsgaard et al., 2004 |
|  |  | communesins G and H | alkaloids | Dalsgaard et al., 2005a |
|  | *P. algidum* | psychrophilin D | cyclic peptide | Dalsgaard et al., 2005b |
| Livingston Island, Antarctica | *Tritirachium* sp. | dimethyldiphenylether A | diphenylether | Ivanova et al., 2007 |
|  |  | macrosphelide A and J | macrotriolides |  |
| King George Island, Antarctica | *Geomyces* sp. | ethyl asterrate; n-butyl asterrate; geomycins A-C | asterric acid derivatives | Li et al., 2008 |
|  | *Trichoderma asperellum* | asperelines A-F | peptaibols | Ren et al., 2009 |
|  | *Oidiodendron truncatum* | chetracins B and C | epipolythiodioxopiperazines | Li et al., 2012a |
|  |  | chetracin D; oidioperazines A−D | diketopiperazines |  |
|  | *Pseudogymnoascus* sp. | pseudogymnoascins A–C and 3-nitroasterric acid | nitro asterric acid derivatives | Figueroa et al., 2015 |
| deep-sea | *Phialocephala* sp. | oxosorbiquinol, dihydrooxosorbiquinol | bisorbicillinoids | Li et al., 2007a |
|  |  | trisorbicillinone A | sorbicillin trimer | Li et al., 2007b |
| deep-sea (Pacific Ocean) | *Penicillium* sp. | breviones F-K | breviane spiroditerpenoids | Li et al., 2009; Li et al., 2012b |
|  |  | sterolic acid | diepoxy sterols |  |
| deep-sea (South China Sea) | *Aspergillus westerdijkiae* | circumdatins K and L | benzodiazepine alkaloids | Peng et al., 2013 |
|  |  | 5-chlorosclerotiamide and 10-epi-sclerotiamide | prenylated indole alkaloids |  |
|  |  | aspergilliamide B | amide |  |
| deep-sea | *Penicillium* sp. | meleagrins B-E; roquefortines F-I | alkaloids | Du et al., 2009; Du et al., 2010 ; Guo et al., 2013 |
|  |  | conidiogenones B–G | diterpenes |  |
|  |  | sorbicillamines A−E | nitrogen-containing sorbicillinoids |  |
| deep-sea | *Phialocephala*  sp. | Dihydrotrichodermolide; dihydrodemethylsorbicillin, trisorbicillinones B-D | sorbicillinoids | Li et al., 2010; Li et al., 2011a |
|  |  | phialofurone | benzofuran derivative |  |
| deep-sea | *Penicillium paneum* | penipacids A-E | anthranilic acid derivatives | Li et al., 2011b; Li et al., 2013 |
|  |  | penipanoid A | triazole carboxylic acid |  |
|  |  | penipanoids B and C | quinazolinone alkaloids |  |
| deep-sea | *Penicillium* sp. | penicilliumin A | sesquiterpene quinone | Lin et al., 2012 |
| deep-sea | *Acrostalagmus luteoalbus* | luteoalbusins A–B | indole diketopiperazines | Wang et al., 2012 |
| deep-sea (Antarctica) | *Penicillium crustosum* | penilactones A and B | highly oxygenated polyketides | Wu et al., 2012 |
| deep-sea (Antarctica) | *Penicillium* sp. | eremophilane sesquiterpenes A-E | eremophilane-sesquiterpenes | Lin et al., 2014; Wu et al., 2013 |
|  |  | lactam-type eremophilane A |  |  |
|  |  | chloro-trinoreremophilane sesquiterpene A |  |  |
|  |  | chlorinated eremophilane sesquiterpenes B-E |  |  |
| deep-sea (South China Sea) | *Emericella* sp. | emerixanthones A-D | prenylxanthones | Fredimoses et al., 2014 |
| deep-sea | *Spiromastix* sp. | spiromastixones A−O | chlorodepsidones | Niu et al., 2014 |
| Berkeley Pit lake (acid mine waste), United States | *Penicillium* sp*.* | bisabolane sesquiterpenes | bisabolane sesquiterpenes | Stierle et al., 2004a |
|  |  | 3-alkyl-6,8-dioxy coumarins | coumarins |  |
|  |  | berkelic acid | spiroketal | Stierle et al., 2006 |
|  |  | berkeleydione and berkeleytrione | bicylic sesquiterpenes | Stierle et al., 2004b |
|  | *P. rubrum* | berkeleyones A_C | berkeleyones | Stierle et al., 2011 |
|  | *P. solitum* | berkedrimanes A and B | drimane sesquiterpene lactones | Stierle et al., 2012 |
| housedust (low water activity) | *Aspergillus restrictus and Aspergillus penicilloides* | arestrictins A and B | dioxopiperazine derivatives | Itabashi et al., 2006 |
| ash of volcano Huguangyan, Republic of China. | *P. citrinum* | steroids with bicyclo[4.4.1]A/B rings | C25 steroids | Du et al., 2008 |
| hydrothermal vent, Taiwan | *A. clavatus* | clavatustides A and B | cyclodepsipeptides | Jiang et al., 2013 |
| Jilantai salt field, Mongolia | *A. variecolor* | variecolorquinones A and B | quinone | Wang et al., 2007 |
|  | *P. citrinum* | pennicitrinone C and penicitrinol B | citrinin dimers | Lu et al., 2008 |
| Hongdao sea salt field, Republic of China | *Alternaria raphani* | alternarosides A-C | cerebrosides | Wang et al., 2009 |
|  |  | alternarosin A | diketopiperazine alkaloid |  |
| Putian salt field, Republic of China | *Aspergillus sclerotiorum* | (S,E)-3-methyl-2-(Nmethylacetamido)-N-(2-(7-(3-methylbut-2-enyl)-1H-indol-3-yl)vinyl) butanamide | indole-3-ethenamide | Wang et al., 2011 |
| Salina, Republic of China | *Myrothecium sp.* | N-acetyl-2,4,10,17-tetrahydroxyheptadecylamine (1) and N-acetyl-3,5,11,18-tetrahydroxyoctadecyl-2-amine | amides | Liu et al., 2015 |

**References**

Bashyal, B.P., Wijeratne, E.M.K., Faeth, S.H., Gunatilaka A.A.L. (2005). Globosumones A-C, cytotoxic orsellinic acid esters from the Sonoran Desert endophytic fungus *Chaetomium globosum*. *J. Nat. Prod.* 68, 724-728.

Chu, Y-S., Niu, X-M., Wang, Y-L., Guo, J-P., Pan, W-Z., Huang, X-W., Zhang, K-Q. (2010). Isolation of putative biosynthetic intermediates of prenylated indole alkaloids from a thermophilic fungus *Talaromyces thermophilus*. *Org. Lett.* 12, 4356-4359.

Dalsgaard, P.W., Blunt, J. W.,. Munro, M. H. G., Larsen, T. O., Christophersen, C. (2004). Psychrophilin B and C: cyclic nitropeptides from the psychrotolerant fungus *Penicillium rivulum*. *J. Nat. Prod.* 67, 1950-1952.

Dalsgaard, P.W., Blunt, J. W,. Munro, M. H. G., Frisvad, J. C., Christophersen, C. (2005a). Communesins G and H, new alkaloids from the psychrotolerant fungus *Penicillium rivulum*. *J. Nat. Prod.* 68, 258-261.

Dalsgaard, P.W., Larsen, T. O., Christophersen, C. (2005b). Bioactive cyclic peptides from the psychrotolerant fungus *Penicillium algidum*. *J. Antibiot.* 58: 141–144.

Du, L., Zhu, T., Fang, Y., Gu, Q., Zhu, W. (2008). Unusual C25 steroid isomers with bicyclo[4.4.1]A/B rings from a volcano ash-derived fungus *Penicillium citrinum.* *J. Nat. Prod.* 71, 1343–1351.

Du, L., Li, D., Zhu, T., Cai, S., Wang, F., Xiao, X., Gu, Q. (2009). New alkaloids and diterpenes from a deep ocean sediment derived fungus *Penicillium* sp. *Tetrahedron* 65, 1033–1039.

Du, L., Feng, T., Zhao, B., Li, D., Cai, S., Zhu, T., *et al*. (2010). Alkaloids from a deep ocean sediment-derived fungus *Penicillium* sp. and their antitumor activities. *J. Antibiot.* 63, 165–170.

[Figueroa, L](http://www.ncbi.nlm.nih.gov/pubmed/?term=Figueroa L%5BAuthor%5D&cauthor=true&cauthor_uid=25732560)., [Jiménez, C](http://www.ncbi.nlm.nih.gov/pubmed/?term=Jiménez C%5BAuthor%5D&cauthor=true&cauthor_uid=25732560)., [Rodríguez, J](http://www.ncbi.nlm.nih.gov/pubmed/?term=Rodríguez J%5BAuthor%5D&cauthor=true&cauthor_uid=25732560)., [Areche, C](http://www.ncbi.nlm.nih.gov/pubmed/?term=Areche C%5BAuthor%5D&cauthor=true&cauthor_uid=25732560)., [Chávez, R](http://www.ncbi.nlm.nih.gov/pubmed/?term=Chávez R%5BAuthor%5D&cauthor=true&cauthor_uid=25732560)., [Henríquez, M](http://www.ncbi.nlm.nih.gov/pubmed/?term=Henríquez M%5BAuthor%5D&cauthor=true&cauthor_uid=25732560)., *et al*. (2015). 3-Nitroasterric acid derivatives from an Antarctic sponge-derived *Pseudogymnoascus* sp. fungus. [*J. Nat. Prod.*](http://www.ncbi.nlm.nih.gov/pubmed/25732560)  78, 919-923.

Fredimoses, M., Zhou, X., Lin, X., Tian, X., Ai, W., Wang, J., *et al*. (2014). New prenylxanthones from the deep-dea derived fungus *Emericella* sp. SCSIO 05240. *Mar. Drugs*, 12, 3190-3202.

Guo, J-P., Tan, J-L., Wang, Y-L., Wu, H-Y., Zhang, C-P., Niu, X-M., *et al*. (2011). Isolation of talathermophilins from the thermophilic fungus *Talaromyces thermophilus* YM3-4. *J. Nat. Prod.* 74, 2278−2281.

Guo, J-P., Zhu, C-Y., Zhang, C-P., Chu, Y-S., Wang, Y-L., Zhang, J-X., *et al*. (2012). Thermolides, potent nematocidal PKS-NRPS hybrid metabolites from thermophilic fungus *Talaromyces thermophilus*. *J. Am. Chem. Soc.* 134, 20306−20309.

Guo, W., Peng, J., Zhu, T., Gu, Q., Keyzers, R. A., Li, D. (2013). Sorbicillamines A−E, nitrogen-containing sorbicillinoids from the deep-sea-derived fungus *Penicillium* sp. F23−2. *J. Nat. Prod*. 76, 2106−2112.

He, J., Wijeratne, E.M.B., Bashyal, B.P., Zhan, J., Seliga, C.J., Liu, M.X., *et al*. (2004). Cytotoxic and other metabolites of *Aspergillus* inhabiting the rhizosphere Sonoran Desert plants. *J. Nat. Prod.* 67, 1985-1991.

Itabashi, T., Matsuishi, N., Hosoe, T., Toyazaki, N., Udagawa, S-i., Imai, T., *et al*. (2006). Two new dioxopiperazine derivatives, arestrictins A and B, isolated from *Aspergillus restrictus* and *Aspergillus penicilloides*. *Chem. Pharm. Bull.* 54, 1639-1641.

Ivanova, V., Kolarova, M., Aleksieva, K. (2007). Diphenylether and macrotriolides occurring in a fungal isolate from the Antarctic lichen *Neuropogon.* *Prep. Biochem. Biotechnol.* 37, 39–45.

Jiang, W., Ye, P., Chen, C-T.A., Wang, K., Liu, P., He, S., *et al*. (2013). Two novel hepatocellular carcinoma cycle inhibitory cyclodepsipeptides from a hydrothermal vent crab-associated fungus *Aspergillus clavatus* C2WU. *Mar. Drugs* 11, 4761-4772.

Li, D., Wang, F., Cai, S., Zeng, X., Xiao, X., Gu, Q., Zhu,W. (2007a). Two new bisorbicillinoids isolated from a deep-sea fungus, *Phialocephala* sp. FL30r. *J. Antibiot.* 60, 317–320.

Li, D., Wang, F., Xiao, Fang, Y.,Zhu, T., Gua, Q., Zhu, W. (2007b). Trisorbicillinone A, a novel sorbicillin trimer, from a deep sea fungus, *Phialocephala* sp. FL30r. *Tetrahedron Lett.* 48, 5235–5238.

Li, Y., Sun, B., Liu, S., Jiang, L., Liu, X., Zhang, H., Che, Y. (2008). Bioactive asterric acid derivatives from the Antarctic ascomycete fungus *Geomyces* sp. *J. Nat. Prod.* 71, 1643–1646.

Li, Y., Ye, D., Chen, X., Lu, X., Shao, Z., Zhang, H., Che, Y. (2009). Breviane spiroditerpenoids from an extreme-tolerant *Penicillium* sp. isolated from a deep sea sediment sample. *J. Nat. Prod.* 72, 912–916.

Li, D., Cai, S., Zhu, T., Wang, F., Xiao, X., Gu, Q. (2010). Three new sorbicillin trimers, trisorbicillinones B, C, and D, from a deep ocean sediment derived fungus, *Phialocephala* sp. FL30r. *Tetrahedron* 66, 5101-5106.

Li, D-H., Cai, S-X., Zhu, T.-J., Wang, F-P., Xiao, X., Gu Q-Q. (2011a). New cytotoxic metabolites from a deep-sea-derived fungus, *Phialocephala* sp., strain FL30r. *Chem. Biodiversity* 8, 895-901.

Li, C-S., An, C-Y., Li, X-M., Gao, S-S., Cui, C-M., Sun, H-F., Wang, B-G. (2011b).|Triazole and dihydroimidazole alkaloids from the marine sediment-derived fungus *Penicillium paneum* SD-44. *J. Nat. Prod.* 74, 1331–1334.

Li, L., Li, D., Luan, Y., Gu, Q., Zhu, T. (2012a). Cytotoxic metabolites from the Antarctic psychrophilic fungus *Oidiodendron truncatum*. *J. Nat. Prod.* 75, 920−927.

Li, Y., Ye, D., Shao, Z., Cui, C., Che, Y. (2012b). A sterol and spiroditerpenoids from a *Penicillium* sp. isolated from a deep sea sediment sample*. Mar. Drugs,* 10, 497-508.

Li, C-S., Li, X-M., Gao, S-S., Lu, Y-H., Wang, B-G. (2013). Cytotoxic anthranilic acid derivatives from deep sea sediment-derived fungus *Penicillium paneum* SD-44. *Mar. Drugs*. 11, 3068-3076.

Lin, X., Zhou, X., Wang, F., Liu, K.(2012). A new cytotoxic sesquiterpene quinone produced by *Penicillium* sp. F00120 isolated from a deep sea sediment sample. *Mar. Drugs.* 10, 106-115.

[Lin, A](http://www.ncbi.nlm.nih.gov/pubmed/?term=Lin A%5BAuthor%5D&cauthor=true&cauthor_uid=24078435)., [Wu, G](http://www.ncbi.nlm.nih.gov/pubmed/?term=Wu G%5BAuthor%5D&cauthor=true&cauthor_uid=24078435)., [Gu, Q](http://www.ncbi.nlm.nih.gov/pubmed/?term=Gu Q%5BAuthor%5D&cauthor=true&cauthor_uid=24078435)., [Zhu, T](http://www.ncbi.nlm.nih.gov/pubmed/?term=Zhu T%5BAuthor%5D&cauthor=true&cauthor_uid=24078435)., [Li, D](http://www.ncbi.nlm.nih.gov/pubmed/?term=Li D%5BAuthor%5D&cauthor=true&cauthor_uid=24078435). (2014). New eremophilane-type sesquiterpenes from an Antarctic deep-sea derived fungus*, Penicillium* sp. PR19 N-1. [*Arch. Pharm. Res.*](http://www.ncbi.nlm.nih.gov/pubmed/24078435) 37, 839-844.

Liu, T., Zhang, S., Zhu, J., Pan, H., Bai, J., Li, Z., *et al*. (2015). Two new amides from a halotolerant fungus *Myrothecium* sp. GS-17. *J. Antibiot.* 68, 267–270.

Lu, Z-Y., Lin, Z-J., Wang, W-L., Du, L., Zhu, T-J., Fang, Y-C., *et al*. (2008). Citrinin dimers from the halotolerant fungus *Penicillium citrinum* B-57. *J. Nat. Prod.* 71, 543–546.

Niu, S., Liu, D., Hu, X., Proksch, P., Shao, Z., Lin, W. (2014). Spiromastixones A−O, antibacterial chlorodepsidones from a deep-sea-derived *Spiromastix* sp. fungus. *J. Nat. Prod.* 77, 1021−1030.

Peng, J., Zhang, X-Y., Tu, Z-C., Xu, X-Y., Qi, S-H. (2013). Alkaloids from the deep-sea-derived fungus *Aspergillus westerdijkiae* DFFSCS013. *J. Nat. Prod.* 76, 983−987.

Ren, J., Xue, C., Tian, L., Xu, M., Chen, J., Deng, Z., *et al*. (2009). Asperelines A-F, peptaibols from the marine-derived fungus *Trichoderma asperellum*. *J. Nat. Prod.*72, 1036–1044.

Stierle, A.A., Stierle, D.B., Kemp, K. (2004a). Novel sesquiterpenoid matrix metalloproteinase-3 inhibitors from an acid mine waste extremophile. *J. Nat. Prod.* 67, 1392-1395.

[Stierle](http://pubs.acs.org/action/doSearch?ContribStored=Stierle%2C+D+B) , D. B., [Stierle](http://pubs.acs.org/action/doSearch?ContribStored=Stierle%2C+A+A), A.A., [Hobbs](http://pubs.acs.org/action/doSearch?ContribStored=Hobbs%2C+J+D), J.D., [Stokken](http://pubs.acs.org/action/doSearch?ContribStored=Stokken%2C+J), J.,Clardy, J. (2004b). Berkeleydione and berkeleytrione, new bioactive metabolites from an acid mine organism. Org. Lett. 6, 1049–1052.

Stierle, A. A., Stierle, D. B., Kelly, K. (2006). Berkelic acid, a novel spiroketal with selective anticancer activity from an acid mine waste*. J. Org. Chem.* 71, 5357- 5360.

Stierle, D.B., Stierle, A.A., Patacini, B., McIntyre, K., Girtsman, T., Bolstad, E. (2011). Berkeleyones and related meroterpenes from a deep water acid mine waste fungus that inhibit the production of interleukin 1-β from induced inflammasomes. *J. Nat. Prod.* 74, 2273–2277.

Stierle, D.B., Stierle, A.A., Girtsman, T., McIntyre, K., Nichols, J. (2012). Caspase-1 and -3 inhibiting drimane sesquiterpenoids from the extremophilic fungus *Penicillium solitum*. *J. Nat. Prod.* 75, 262−266.

Wang, W., Zhu, T., Tao, H., Lu, Z., Fang, Y., Gu, Q., Zhu, W. (2007). Two new cytotoxic quinone type compounds from the halotolerant fungus *Aspergillus variecolor*. *J. Antibiot.* 60, 603–607.

Wang, W., Wang, Y., Tao, H., Peng, X., Liu, P., Zhu, W. (2009). Cerebrosides of the halotolerant fungus *Alternaria raphani* isolated from a sea salt field. *J. Nat. Prod.* 72, 1695–1698.

Wang, H., Zheng, J-K., Qu, H-J., Liu, P-P., Wang, Y., Zhu, W-M. (2011). A new cytotoxic indole-3-ethenamide from the halotolerant fungus *Aspergillus sclerotiorum* PT06-1. [*J Antibiot (Tokyo).*](http://www.ncbi.nlm.nih.gov/pubmed/21792207) 64, 679-681.

Wang, F-Z., Huang, Z., Shi, X-F., Chen, Y-C., Zhang, W-M., Tian, X-P., *et al*. (2012). Cytotoxic indole diketopiperazines from the deep sea-derived fungus *Acrostalagmus luteoalbus* SCSIO F457. *Bioorg. Med. Chem. Lett.* 22, 7265–7267.

Wu, G., Ma, H., Zhu, T., Li, J., Gu, Q., Li, D. (2012). Penilactones A and B, two novel polyketides from Antarctic deep-sea derived fungus *Penicillium crustosum* PRB-2. *Tetrahedron* 68, 9745-9749.

Wu, G., Lin, A., Gu, Q., Zhu, T., Li, D. (2013). Four new chloro-eremophilane sesquiterpenes from an Antarctic deep-sea derived fungus, *Penicillium* sp. PR19N-1. *Mar. Drugs*, 11, 1399-1408.

Yang, Y-L., Liao, W-Y., Liu, W-Y., Liaw, C.-C., Shen, C-N., Huang, Z-Y., Wu, S-H. (2009). Discovery of new natural products by intact-cell mass spectrometry and LC-SPE-NMR: malbranpyrroles, novel polyketides from thermophilic fungus *Malbranchea sulfurea*. *Chem. Eur. J.* 15, 11573 - 11580.
